# The Effect of Double Carbapenem Regimen in the Management of Carbapenem-Resistant Klebsiella pneumoniae Infections: A Report of Five Cases

**DOI:** 10.7759/cureus.52023

**Published:** 2024-01-10

**Authors:** Radhika Soanker, Lakshmi Vemu, Sukanya Vimala, Padmaja Kanne, Venu M Thumma, Gayatri Chavala

**Affiliations:** 1 Pharmacology, All India Institute of Medical Sciences, Bibinagar, Hyderabad, IND; 2 Microbiology, Kamineni Academy of Medical Sciences and Research Centre, Hyderabad, IND; 3 Microbiology, Nizam's Institute of Medical Sciences, Hyderabad, IND; 4 Surgical Gastroenterology, Nizam's Institute of Medical Sciences, Hyderabad, IND; 5 Clinical Pharmacology and Therapeutics, Nizam's Institute of Medical Sciences, Hyderabad, IND

**Keywords:** pan-drug resistant, extensively-drug resistant, difficult to treat pathogens, k. pneumoniae producing carbapenemase, metallo beta-lactamase

## Abstract

The treatment of infections caused by carbapenem-resistant organisms is challenging. Carbapenems in combination with vaborbactam and relebactam are recommended to treat infections caused by extensively drug-resistant organisms including carbapenemase-producing isolates, while ceftazidime-avibactam plus aztreonam, or cefiderocol is recommended for infections caused by New Delhi metallo beta-lactamase (NDM)-producing *Enterobacteriaceae*. As, in India, except for ceftazidime-avibactam and aztreonam, the other drugs are not approved for marketing, in this case report, the role of a double carbapenem regimen (ertapenem plus meropenem) in the treatment of carbapenem-resistant *Klebsiella pneumoniae* infections has been presented. In one case, the in vitro effect of the double carbapenem regimen on pan drug-resistant (PDR) *K. pneumoniae* isolates from a blood culture specimen of a critically ill patient using a time-kill study is presented. In this case, only a double carbapenem regimen with 2 MIC (minimum inhibitory concentration) meropenem + 2 MIC ertapenem demonstrated bactericidal activity by inhibition of bacterial growth of PDR *K. pneumoniae* isolate, at four and eight hours, which was sustained till 24 hours. However, while 2 MIC meropenem + 2 MIC colistin inhibited bacterial growth at four hours and eight hours, bacterial regrowth occurred by 24 hours. In addition, four cases of critically ill patients with infections caused by carbapenem-resistant *Enterobacteriaceae *are presented in whom a double carbapenem regimen was recommended for treatment. Of these four cases, a complete clinical cure was observed in three cases, and a microbiological cure in the fourth case. As the double carbapenem regimen demonstrated effect in an in vitro time-kill study in the first case and on clinical outcomes in three out of the latter four cases, it appears to be a life-saving, salvage therapy in infections caused by carbapenem-resistant* K. pneumoniae *in India.

## Introduction

Acquisition of resistance by pathogenic bacteria to multiple antimicrobial agents has become a great menace to mankind. The treatment of infections caused by carbapenem-resistant organisms is challenging, as there are very few therapeutic options. 

When an organism is resistant to all the antimicrobials in all anti-microbial classes, it is referred to as pan-drug resistant (PDR). An organism that is resistant to at least one antimicrobial in all antimicrobial classes except for two or fewer antimicrobial classes is referred to as extensively drug-resistant [1,2]. *Enterobacteriaceae* that test resistant to at least one of the carbapenem antibiotics or produce a carbapenemase are called carbapenem-resistant *Enterobacteriaceae *(CRE) [3].

In the United States, infections with CREs are associated with hospitalization, and one-third of CREs are *Klebsiella pneumoniae* carbapenemase (KPC) producers. New Delhi metallo β-lactamase (NDM), Verona Integron-encoded metallo-β-lactamase (VIM), and imipenemase (IMP) producers are less reported in the United States. Among the CREs identified, only 1/10th were metallo-β-lactamases (MBL) producers [3].

Indian Council of Medical Research (ICMR) in its 2021 report on antimicrobial resistance (AMR) research and surveillance network across India from 39 tertiary care centers delineates that between 2016 and 2021, imipenem susceptibility of *Escherichia coli* decreased from 86% to 64%, while that of *Klebsiella pneumoniae* decreased from 65% to 43% [1]. A very high rate of meropenem resistance was reported in 61% of *Providencia rettgeri*, followed by 55% in *K. pneumoniae*, and 36% in *Klebsiella oxytoca*, while susceptibility was reported to be 60% among *Enterobacteriaceae*. Of the 209 *K. pneumoniae*-resistant isolates, 40% were NDM, 39% were OXA-48, and 15% were KPC. Of the 279 E*. coli*-resistant isolates, 31% were NDM and 5% were KPC [4].

As CRE are resistant to β-lactam antibiotics including carbapenems [5], they may be intermediate/susceptible to polymixins, tigecycline, fosfomycin, gentamicin, and cotrimoxazole. Although clinical outcomes with a combination of two or more in vitro-active drugs were reported to be superior to treatment with one in vitro-active drug in cases with proven CRE infections, the rates of treatment failure also remain high [6,7].

ICMR recommends for complicated infections or hemodynamically unstable patients with CREs either a combination of polymyxins with an antimicrobial agent with proven in vitro susceptibility or based on the susceptibility of isolate, ceftazidime-avibactam alone or ceftazidime-avibactam with aztreonam or high-dose carbapenems [8]. However, empiric use of aztreonam plus ceftazidime/avibactam combination therapy is not recommended and is recommended only when susceptibility is proven to this regimen [8]. In addition, combination therapy is very expensive and all the laboratories may not support in vitro synergy tests for this combination.

Owing to high resistance to carbapenems in *Enterobacteriaceae* isolates, the 14-day mortality in cases of bloodstream infections (BSI) and cases of urinary tract infections (UTI) was observed to be 38.1% and 27.9%, respectively, and of the total BSI cases and total UTI cases, only 10% and 11.4% were discharged on Day 14, respectively [4].

In this prevailing situation, there is an urgent need to assess combination regimens that can be effective in the treatment of CRE infections. The Western literature and Indian consensus on the management of CRE infections propose the addition of ertapenem to either meropenem, doripenem, or imipenem alone or in combination with colistimethate sodium as a rescue or salvage treatment for carbapenem-resistant *K. pneumoniae* (CR-Kp) infections [5,9-15].

The combination therapy, also known as double carbapenem regimen, was based on the observation that the percentage of CR-Kp isolates susceptible to ertapenem was less, which can be explained by the significant affinity between ertapenem and carbapenemase. Therefore, in the proposed regimen, ertapenem will play the role of suicide antimicrobial thereby allowing the most active carbapenem to exert maximum inhibitory effect on the isolate [5].

Although the efficacy of a double carbapenem regimen is reported in in vitro studies, case reports, short case series, and some retrospective and prospective observational studies from Western countries [5,11,13-15], its relevance in the treatment of complicated infections with carbapenem-resistant enterobacterales in India is still unexplored [13] to the extent that it did not even merit mention in the latest ICMR guidelines related to the management of infections caused by carbapenem-resistant isolates in 2022 [8].

In India, as there are not many treatment options for the management of CR-Kp infections and as the gene blaNDM is reported to contribute to resistance to carbapenems in contrary to blaKPC in Western countries, this case report is being presented to highlight the effect of double carbapenem regimen for PDR *K. pneumoniae* isolates in an in vitro time-kill study and its role in the treatment of patients with CR-Kp infections.

## Case presentation

All the cases presented here were referred to Drug Information Services (DIS) provided by the Department of Clinical Pharmacology and Therapeutics. These services were offered to clinicians. Clinicians referred cases for opinion when they suspected drug-related therapeutic issues like the selection of antibiotics based on culture/susceptibility report, dosage adjustment in special populations, drug-drug interactions, and adverse drug reactions and/or rechallenge options.

In vitro efficacy of double carbapenem regimen

Case 1

A case was referred to DIS from an intensive care unit for an opinion on the antibiotic to be prescribed based on the culture susceptibility report. A pan-drug-resistant isolate of *K. pneumoniae* was reported in the blood culture/susceptibility report. VITEK® system (bioMérieux SA, Marcy-l'Étoile, France) was used for the identification of the isolate from the blood sample and antimicrobial susceptibility testing.

As there were no standard treatment guidelines for the treatment of infections caused by PDR organisms, a time-kill study for assessment of the effect of a double carbapenem regimen (meropenem + ertapenem) on this PDR isolate was performed. An in vitro assay was conducted, in which the minimum inhibitory concentrations (MICs) of meropenem, ertapenem, and colistin were estimated using broth macrodilution method and checkerboard test with a bacterial inoculum of 5 X 10^5^ CFU/ml of two clinical isolates, one PDR *K. pneumoniae*, and one colistin-susceptible CR-Kp. MICs of 12 mcg/mL of meropenem, 6 mcg/mL of ertapenem, and 6 mcg/mL of colistin for PDR *K. pneumoniae* were obtained.

Based on the checkerboard synergy test results, the activities of the different antimicrobial combinations as enlisted in Table 1, were assessed in the time-kill study.

**Table 1:** Different combination of antimicrobials used for time-kill assay MIC: minimum inhibitory concentration

S.No	Combination of Antimicrobials
1	Growth Control
2	0.5 MIC Meropenem + 0.5 MIC Colistin
3	1 MIC Meropenem + 1 MIC Colistin
4	2 MIC Meropenem + 2 MIC Colistin
5	0.5 MIC Meropenem + 0.5 MIC Ertapenem
6	1 MIC Meropenem + 1 MIC Ertapenem
7	2 MIC Meropenem + 2 MIC Ertapenem
8	0.5 MIC Ertapenem + 0.5 MIC Colistin
9	1 MIC Ertapenem + 1 MIC Colistin
10	2 MIC Ertapenem + 2 MIC Colistin
11	1 MIC Meropenem + 1 MIC Ertapenem + 1MIC Colistin

The time-kill study was conducted in the logarithmic growth phase using an initial bacterial inoculum of 5 X 10^5^ CFU/mL. The bacterial inoculum of each of the test isolates was added to each of the drug combinations prepared in separate tubes and thoroughly mixed. Two growth control tubes, without any antimicrobial agent, were also included. All the tubes were incubated at 37 degrees C.

The test isolates were then spot inoculated on 5% sheep blood agar plates (COS plates; bioMérieux SA, Marcy-l'Étoile, France) at zero hours, four hours, eight hours, and 24 hours of incubation. All of them were incubated at 37 degrees C for 24 hours. The log CFU/mL of bacterial growth at four, eight, and 24 hours was documented for different combinations of drugs. The anti-microbial activity of different combinations as enlisted in Table 1 was thus investigated by a time-kill study. The bactericidal effect was defined as a ≥ 3-log_10_ CFU/mL decrease at 24 hours from the initial inoculum.

On the growth control plate, bacterial growth had increased by 5-log_10_ CFU/mL at 24 hours for both isolates.

Time-kill study of colistin-sensitive CR-Kp isolate revealed that four different combinations, 2 MIC meropenem + 2 MIC ertapenem; 1 MIC meropenem + 1 MIC ertapenem; 2 MIC meropenem + 2 MIC colistin; and 1 MIC meropenem + 1 MIC ertapenem + 1 MIC colistin inhibited the bacterial growth at four and eight hours, which was sustained till 24 hours. Of these, 1 MIC meropenem + 1 MIC colistin inhibited the growth at four hours; however, regrowth occurred at eight hours and growth increased at 24 hours. None of the other combinations demonstrated bacterial growth inhibition. These observations suggest that the double carbapenem regimen (meropenem plus ertapenem) in 1 or 2 MIC with or without colistin was effective in inhibiting the growth of the isolate while meropenem + colistin, even though effective in a 2 MIC combination, did not demonstrate a similar effect in 1 MIC combination.

In the time-kill study of PDR *K. pneumoniae* isolate, only the double carbapenem regimen with 2 MIC meropenem + 2 MIC ertapenem demonstrated bactericidal activity by inhibition of bacterial growth at four and eight hours, which was sustained till 24 hours; 2 MIC meropenem + 2 MIC colistin inhibited the bacterial growth at four hours and eight hours; however, bacterial regrowth occurred by 24 hours. On the other hand, 1 MIC meropenem + 1 MIC ertapenem demonstrated a gradual increase in growth inhibition from four hours to 24 hours, attaining complete inhibition of growth at 24 hours, and 1 MIC ertapenem + 1 MIC meropenem + 1 MIC colistin inhibited the bacterial growth at four hours, but regrowth appeared at eight hours, which gradually increased at 24 hours. None of the other combinations tested inhibited bacterial growth. These results have demonstrated that only a double carbapenem regimen (meropenem plus ertapenem) in 2 or 1 MIC was effective in inhibiting PDR isolate.

Effect of double carbapenem regimen on clinical outcomes

Four cases (Cases A-D) were referred from intensive care units to DIS for advice on antibiotic therapeutic options based on the culture/susceptibility reports. The median age of these four cases was 32.5 years. Of them, two were male and two female. One case had sepsis secondary to lower respiratory tract infection and the remaining three cases had developed postoperative intra-abdominal infection. Cultures reported PDR *K. pneumoniae* in two of the four cases and colistin-sensitive CR-Kp in the other two cases. A double carbapenem regimen with meropenem and ertapenem was advised in all four cases. The patients were monitored for microbiological and clinical cures. Complete recovery from the infection was treated as a clinical cure and no growth of index pathogen in the serial cultures repeated from the index site was treated as a microbiological cure. The median duration of treatment with a double carbapenem regimen was observed to be 16 days (10-35 days). The median duration to clinical cure was determined to be 20 days. All four cases tolerated the double carbapenem regimen well. The case details are given below.

Case A

A female patient aged 83 years, with co-morbidities of parkinsonism, diabetes, and hypertension, was admitted to the critical care unit for suspected pneumonia. She was empirically treated with meropenem 500 mg IV every 12 hours and colistin 1.5 MIU IV every eight hours (renal adjusted dosage). The blood culture reported PDR *K. pneumoniae*. The case was referred to DIS for therapeutic options.

Double carbapenem regimen was recommended with meropenem 500 mg IV every 12 hours and ertapenem 500 mg every 24 hours (renal adjusted dose). The clinicians continued colistin. A repeat blood culture after 48 hours of therapy was positive for PDR *K. pneumoniae* and on Day 5, the blood culture was reported sterile indicating microbiological cure. The regimen was continued until day 10.

On day 10, the culture of tracheal aspirate reported carbapenem-resistant, colistin-sensitive *Klebsiella ozaenae*. Given this, the clinicians stopped the double carbapenem regimen and colistin due to a lack of substantial evidence to support a double carbapenem regimen. Alternatively, the patient was prescribed polymixin B. Subsequently, the total leucocyte count rose and the blood culture on Day 14 reported PDR *K. pneumoniae*, and subsequently the patient died.

Case B

A female aged 25 years was admitted to the surgical gastroenterology ward with a diagnosis of a complicated choledochal cyst. She had a history of cholangitis for which she required repeated biliary stenting. She underwent cyst excision and hepaticojejunostomy. Postoperatively, she required reexploration and peritoneal lavage for biliary leak and collection. After ordering the cultures, meropenem 1g every eight hours IV was given empirically along with clindamycin, metronidazole, and fluconazole. Cultures of abdominal pus reported *Acinetobacter baumanii* while blood culture reported PDR *K. pneumoniae*. Colistin 3 MIU every eight hours was added to the regimen. After six days of adding colistin, as the condition of the patient was worsening, the case was referred to drug information services for an opinion on appropriate therapy.

DIS advised a double carbapenem regimen with meropenem 1g every eight hours and ertapenem 1g every 24 hours for *K. pneumoniae*. It suggested cefoperazone-sulbactam (2g/2g) every 12 hours for *A. baumanii* based on the susceptibility report. On day 4, the clinical condition of the patient improved and total bilirubin dropped from 4.6 mg/dl to 3.7 mg/dl. On day 12, the patient became afebrile, and the double carbapenem regimen and cefoperazone-sulbactam were stopped, indicating a clinical cure, and the patient was discharged in a stable condition. On the follow-up visit on day 26, the patient was in good condition.

Case C

A male aged 40 years was admitted with a diagnosis of choledocholithiasis. He had a history of cholangitis for which he underwent endoscopic retrograde cholangiopancreatography (ERCP) and biliary stenting. He underwent common bile duct exploration with clearance of stones and T-tube drainage of the bile duct and was transferred to the critical care unit for postoperative high-grade fever. Bile culture was ordered. Empiric treatment was started with meropenem, colistin, linezolid, and metronidazole. Bile culture reported colistin-sensitive CR-Kp. The case was referred to DIS for an opinion on therapeutic options. DIS suggested a double carbapenem regimen with meropenem 1g every eight hours and ertapenem 1g every 24 hours. However, the clinician continued colistin, linezolid, and metronidazole. Metronidazole was stopped after a week.

Drain fluid culture, ordered on day 2, reported *K. pneumoniae* resistant to colistin and carbapenems with susceptibility to tigecyline and aminoglycosides. Clinicians continued the double carbapenem regimen at the recommendation of DIS and continued colistin and linezolid. The clinical condition of the patient showed improvement on day 10 with decreasing spikes of fever. On day 20, the fever subsided, the clinical condition improved, and all antimicrobials including the double carbapenem regimen were stopped indicating a clinical cure. The patient was discharged in stable condition on day 22.

Case D

A male aged 21 years underwent distal pancreatectomy and splenectomy for Grade III pancreatic trauma. Abdominal pus culture and peritoneal fluid cultures were ordered. He was shifted to the ICU with a high-grade fever. He was started on meropenem, colistin, linezolid, and fluconazole as empiric treatment. Both cultures reported colistin-sensitive CR-Kp. The case was referred to DIS for an opinion.

At the advice of DIS, a double carbapenem regimen with meropenem 1g every eight hours and ertapenem 1g every 24 hours was added. The clinician continued colistin, metronidazole, and linezolid. A blood culture ordered on day 3 reported *Staphyllococcus hemolyticus*, which was sensitive to vancomycin, teicoplanin, and daptomycin but resistant to linezolid. Subsequently, linezolid was stopped and the patient was started on vancomycin. Repeat drain culture on day 13 reported *Stenotrophomonas maltophilia* sensitive to cotrimoxazole. Cotrimoxazole was prescribed in addition to the ongoing treatment. On day 18, the patient had signs of improvement with a decrease in fever spikes and leucocyte counts. Drain fluid culture ordered on day 35 reported no growth and the patient was discharged in good condition on day 38.

## Discussion

In Case 1, the effect of the double carbapenem regimen in both colistin-sensitive and colistin-resistant CR-Kp isolate cultures was demonstrated by the time-kill study. Based on these results, the in vivo effect of the double carbapenem regimen on clinical outcomes was observed. Also, in three of the four latter cases (Cases A-D), a clinical cure was obtained. These findings support recent literature reporting the clinical efficacy of a double carbapenem regimen in the management of CRE infections caused by KPC strains.

Two bacterial isolates, colistin-sensitive and colistin-resistant CR-Kp isolates, cultured from critically ill patients were tested to understand the efficacy of a double carbapenem regimen in PDR isolates and to test whether this regimen works in Indian settings, where NDM [8] is reported as a mechanism of resistance in a significant percentage of CRE isolates.

It was encouraging to observe that the time-kill study on PDR isolate of *K. pneumoniae* demonstrated antimicrobial activity of a double carbapenem regimen with 2 MIC meropenem + 2 MIC ertapenem by inhibition of bacterial growth at four and eight hours, which was sustained till 24 hours. However, although 2 MIC meropenem + 2 MIC colistin inhibited bacterial growth at four hours and eight hours, bacterial regrowth occurred by 24 hours. This could be one of the reasons for increasing clinical failure rates of colistin-carbapenem combination regimens, especially in critically ill patients.

It was similarly reported in a case series by Oliva et al., which evaluated the in vitro efficacy of meropenem and ertapenem against PDR *K. pneumoniae* isolates [5]. In the time-kill study, they observed that ertapenem alone exhibited an initial decrease in bacterial growth; however, regrowth was observed at 24 hours. Similar results were observed for meropenem alone, while the combination of meropenem and ertapenem demonstrated anti-bacterial activity at four, six, and eight hours, which was sustained up to 24 hours at concentrations of 0.5, 1, and 2 MIC meropenem plus 1 MIC ertapenem in all patients.

A prospective study done by Oliva et al. evaluated the in vitro activity of ertapenem plus meropenem in 15 patients with CR-Kp infection in whom colistin was not indicated [10]. In this study, the time-kill study demonstrated synergistic and bactericidal activity of both 1 MIC and 2 MIC meropenem plus 1 MIC ertapenem at 24 hours in 87.6% and 100% strains, respectively, with a decrease of log CFU/mL significantly vs other regimens (p <0.0001).

In a case report, Ceccarelli reported similar results for the efficacy of ertapenem and meropenem in a time-kill study using a colistin-resistant KPC-producing CR-Kp isolate [7]. They observed that the combinations ertapenem + doripenem or meropenem at 1 MIC were synergistic after four hours, killing 99.9% of colonies, maintaining the bacterial growth inhibition until 24 hours; 1-log increase in bacterial growth at 24 hours was observed with ertapenem alone while 3-log increase was observed with doripenem alone and meropenem alone.

In the present report, the DIS recommended a double carbapenem regimen with meropenem and ertapenem in four patients, who did not respond to empiric treatment with colistimethate sodium and meropenem. The median duration of treatment with the double carbapenem regimen was observed to be 16 days. The median duration to clinical cure was 20 days. Three patients were successfully treated while one patient died subsequent to stopping the double carbapenem regimen and relapse of infection on day 12.

Similar results were obtained in a study done by Oliva et al., which demonstrated the effect of a double carbapenem regimen in 12 out of 15 critically ill patients suspected to be in sepsis and in whom colistin was to be avoided [6]. The median duration of microbiological and clinical response was reported as three days with an overall treatment response in 12 out of 15 cases and a global mortality of one of 15 cases. The median duration of clinical response was reported as three days, which is much less than that observed in the current report (20 days), which cannot be understood in the absence of knowledge of the mechanism of carbapenem resistance of isolates in this report.

In a 1:2 matched case-control study, done in Italy, it was observed that patients receiving standard treatment based on susceptibility reports had a significantly higher rate of 28-day mortality when compared to patients on a double carbapenem regimen with CR-Kp [9]. In addition, a subgroup analysis of cases infected with carbapenem-resistant colistin-resistant strain revealed high clinical cure and microbiological eradication rates in the double carbapenem group when compared to the best available treatment.

In an observational study done in Rome, a double carbapenem regimen was evaluated against the standard of care treatment based on susceptibility reports in cases infected with CRE, and the authors reported that subjects treated with DC regimen had a higher rate of fifth-day response when compared to controls [10]. They observed these good outcomes despite the higher sepsis rate at baseline in the double carbapenem regimen group.

In addition, four cases in the current report tolerated the double carbapenem regimen well without any increased risk of side effects. Similar positive results have been reported in case reports, where patients with CRE infections were managed successfully with a double carbapenem regimen [5,7]. 

In a case series by Giamarellou et al., similar results were published and the authors opined that in an era where PDR strains are emerging, the strategy of administration of double carbapenems for carbapenemase-producing *K. pneumoniae* (CPKP) infections appears to be revolutionary, as they observed that in two patients in whom PDR* K. pneumoniae* was isolated with high MICs to all carbapenems (>32 mcg/ml), the ertapenem-doripenem regimen employed was effective [11]. To confirm their observation, healthy mice were infected with CPKP isolates having MIC value of 64 mg/liter for doripenem and ertapenem MIC 4 mg/liter, using human simulated dosing regimens of combination regimen versus monotherapy. The results were in support of a double carbapenem regimen.

Infectious Disease Society of America (IDSA) guidance recommends meropenem-vaborbactam, ceftazidime-avibactam, or imipenem-cilastatin-relebactam for infections with KPC-producers [16]. In addition, extended-infusion regimens of carbapenems with or without the addition of a second agent for the treatment of CRE are not recommended, as these regimens were found to be associated with lower survival rates and nephrotoxicity. However, for infections caused by MBL producers, it recommends ceftazidime-avibactam + aztreonam, or cefiderocol monotherapy. Though there are a number of publications reporting the effectiveness of a double carbapenem regimen in the treatment of critically ill patients with CPKP infections, it is unclear why this regimen was not discussed in IDSA guidance.

In an update on therapy for the treatment of carbapenem-resistant infections that reviewed the evidence for the effectiveness of a double-carbapenem regimen, it was opined that it is effective in the treatment of CRE infections based on data; however, most of the isolates causing CRE infections were reported to be KPC-producing *K. pneumoniae*. Therefore, the effect of double-carbapenem regimens in infections caused by MBL-producing CRE is yet to be established. They list a double carbapenem regimen with ertapenem administration ahead of the administration of meropenem or doripenem under a second potential strategy (combination of anti-CRE agents) for the treatment of CRE infections in seriously ill patients [12].

Indian consensus on the management of CRE infections published in 2019 emphasizes that contrary to the Western world, in India, NDM-producing CRE are responsible for the majority of CR infections [13]. As in serious CRE infections, studies are reporting improved outcomes with carbapenem-based combination therapy even when CRE isolates are resistant to colistin, it recommended high-dose carbapenem-based combination therapy if it is caused by isolates with MIC ≤16 mg/L. However, it discouraged meropenem-based combination therapy when the MIC of the isolate exceeds 64 mg/L. The panel opined that there might be a limited utility of double carbapenem therapy in Indian clinical settings as the effect of this regimen was demonstrated in KPC-producing CRE infections, and this effect was also with OXA-48 (class D β-lactamase) in some studies, and none of the studies reported its effect in MBL producing CRE infections [13].

A review article on the therapeutic options of MBL-producing *Enterobactarales* was published in 2021 [14]. Interesting facts on the artefacts of the culture susceptibility tests using agar plates that contain higher zinc concentrations than intracellular levels, especially the routinely used Mueller Hinton broth were presented. It was pointed out that as MBL enzymes are known to hydrolyze carbapenems, especially in the presence of zinc, as zinc catalyzes the enzyme activity. This finding was supported by a study done by Asempa et al., in which NDM and KPC producers appeared to be carbapenem, particularly meropenem, resistant but observed to reduce bacterial growth by 1 log in murine infection models. They showed that the meropenem effect in murine infection models had a good correlation with in vitro susceptibility in zinc-depleted media where the isolates were observed to be carbapenem sensitive [14].

Roujansky et al. reported in vivo efficacy of ertapenem and imipenem in a case with NDM-1 producer [15]. In the study, in vivo efficacy of both ERT and IMP in a case with NDM-producing *E. coli* with culture-proven carbapenem resistance was observed. The survival of mice despite infection with NDM-1 producers that are carbapenem resistant was surprising. The authors opined that this paradoxical finding might be useful in explaining the effect of carbapenem combination therapy in patients reported in various publications.

Though none of the guidelines recommend a double carbapenem regimen, it was recommended by DIS in the cases under this report and was prescribed in four cases solely based on literature and time-kill study results under close supervision by the clinicians as no other alternative therapeutic options were available. Amazingly, in India, where NDM producers are highly prevalent [4], in three of four cases, a clinical cure was obtained and in the fourth case, a microbiological cure was obtained with the administration of a double carbapenem regimen.

In this case report, the efficacy of a double carbapenem regimen with/without colistimethate sodium on clinical outcomes in three out of four patients admitted to critical care units with culture-proven CR-Kp infections was demonstrated with a median time to clinical cure as 20 days. These results assume importance in Indian settings as newer agents, which are recommended by IDSA [16] in the treatment of CRE infections, are not available in India [8], and a double carbapenem regimen can be a cost-effective, life-saving, salvage therapy.

## Conclusions

As a complete clinical cure was observed in three of four cases and in the remaining case too, a microbiological cure was observed, the double carbapenem regimen might serve as a cost-effective, life-saving, salvage therapy in infections caused by CR-Kp, especially in India where recommended newer agents are not available for treatment. However, there is a need to develop a point-of-care in vitro susceptibility test to identify the susceptible CRE isolates to a synergistic combination of meropenem and ertapenem and to test the efficacy of double carbapenem regimen in randomized studies, especially against MBL-producing *Enterobacteriaceae*.
